# Intratumoral heterogeneity and clonal evolution in blood malignancies and solid tumors

**DOI:** 10.18632/oncotarget.20279

**Published:** 2017-08-16

**Authors:** Ignacio Varela, Pablo Menendez, Alejandra Sanjuan-Pla

**Affiliations:** ^1^ Instituto de Biomedicina y Biotecnología de Cantabria, Universidad de Cantabria-CSIC, Santander, Spain; ^2^ Department of Biomedicine, Josep Carreras Leukemia Research Institute, School of Medicine, University of Barcelona, Barcelona, Spain; ^3^ Institució Catalana de Recerca i Estudis Avançats, Barcelona, Spain; ^4^ Red de Investigación Biomédica en Red de Cáncer (CIBERONC), ISCIII, Barcelona, Spain; ^5^ Hematology Research Group, IIS La Fe, Valencia, Spain

**Keywords:** cancer genomics, intratumoral heterogeneity, tumor evolution, oncology practice, bioinformatics

## Abstract

This meeting held at the University of Barcelona in March 2017, brought together scientists and clinicians worldwide to discuss current and future clinico-biological implications of intratumoral heterogeneity (ITH) and subclonal evolution in cancer diagnosis, patient stratification, and treatment resistance in diagnosis, treatment and follow-up. There was consensus that both longitudinal and tumor multi-region studies in matched samples are needed to better understand the dynamics of ITH. The contribution of the epigenome and microenvironment to ITH and subclone evolution remains understudied. It was recommended to combine computational, pathology and imaging tools to study the role of the microenvironment in subclone selection/evolution.

## INTRODUCTION

Dr. Pablo Menéndez, an ICREA professor and the Scientific Director of the Campus Clinic Josep Carreras Leukemia Research Institute, opened the meeting by welcoming everyone and acknowledging the funding bodies for their efforts to “reprogram” Barcelona into a scientific and clinical hub for hemato-oncology research.

Cancers evolve through a dynamic process of clonal expansion, genetic diversification and clonal selection within the adaptive landscapes of tissue ecosystems. The highly variable and dynamic patterns of genetic diversity results in a complex intratumor heterogeneity (ITH), the central topic of the meeting. While therapeutic intervention may destroy specific cancer clones, it inadvertently provides a selective pressure for the expansion of other, genetically distinct, clones. This intraclonal cancer evolution has been attributed to Darwinian evolutionary principles, which may lead to therapeutic failure through the ability of specific clones to become therapy resistant.

ITH and clonal evolution are expected to have significant impact on cancer biology, and the clinical significance of ITH and its temporal evolution and dynamics in response to therapy is anticipated to become increasingly relevant to the way we diagnose, treat and follow-up cancer patients. Emerging questions posed from the outset were: i) Will we ever achieve complete responses using monotherapy, or will polytherapy be necessary to manage ITH-mediated malignancy?; ii) Is it crucial to identify the *bona fide* initiating/driver oncogenic event to develop targeted therapeutic strategies?; iii) Does patient stratification at diagnosis need to be revisited?; iv) Should we apply this emerging knowledge to the way we measure minimal residual disease (MRD) in follow-up studies?, etc.

## AN EVOLUTIONARY PERSPECTIVE OF CANCER

The session highlighted the Darwinian branched model as the current schema to explain cancer evolution and subclone emergence and diversification from the tumor trunk. The lineal evolution model with sequential acquisition of mutations was disregarded [1]. Dr. David Posada (University of Vigo, Spain) described tumor phylogeography and spatial distribution of genetic lineages, contending that ITH should be studied using population genetics techniques. He employed Clomial (Bioconductor), a generative binomial model, to analyze next generation sequencing (NGS) data, and demonstrated how statistical phylogeographic inference from multiple tumor regions can aid to understand the *modo and tempo* of evolution in colorectal tumors. Dr. Andrea Sottoriva (Institute of Cancer Research, Sutton, UK) introduced the concept of neutral evolution to reflect selection-free evolution, where all tumor cells have equal growth rate. He highlighted the importance of understanding ITH in the absence of mutations and selection to distinguish between functional and non-functional heterogeneity [2]. Methods to infer the role of natural selection within tumors were presented by Dr. Christina Curtis (Stanford University, CA, USA). By simulating spatial tumor growth under different evolutionary modes and examining patterns of between-region subclonal genetic divergence from multi-region sequencing data, her group showed that it is feasible to distinguish tumors with strong positive subclonal selection from those evolving neutrally or under weak selection. All speakers raised the challenges of automatic frequency-based methods to directly estimate the evolutionary trajectories from bulk sequencing data.

## MOLECULAR DIAGNOSIS OF ITH

The session discussed how to diagnose ITH using NGS or mass-spectometry-based cytometry approaches. In the context of hematopoietic stem cell (HSC) heterogeneity, Dr. David Kent (Cambridge Institute for Biomedical Research, Cambridge, UK) discussed the impact that the acquisition order of mutations (TET2 and JAK2^V617F^) has on the disease phenotype. In addition, through single-cell gene expression and functional studies on HSCs from a JAK2^V617F^ mouse model, he presented data on an HSC subpopulation with a self-renewal defective signature that can be partly restored in TET2/JAK^V617F^ double-mutant single cells, demonstrating that single-cell approaches are important to deconstruct the molecular network of normal and malignant stem cells [3]. The complexity underlying small cell lung cancer (SCLC) was covered by Dr. Caroline Dive (Manchester Cancer Research UK Center, Manchester, UK), who focused on Patient-Derived Xenografts (PDX) to study SCLC evolution, treatment response and resistance. SCLC presents early metastasis and poor prognosis and most patients have circulating tumor cells (CTCs) [4]. Through vasculogenic mimicry (VM), tumor cells may develop their own blood vessels endowing them with invasive and metastatic potential [5]. Clinically, VM could be targeted for therapeutic intervention in SCLC; however, how VM contributes to ITH and clonal diversification is still being unraveled. From a computational perspective, Dr. Núria López-Bigas (Institute of Biomedical Research, Barcelona, Spain) illustrated how cancer drivers may be identified from mutations coming from NGS in cohort studies. She described different bioinformatics tools and resources generated by her group (OncodriveFM, OncodriveROLE and OncodriveCLUST). She also introduced how to approach mutations in coding and non-coding regions, emphasizing the need to collect biomedical genomic data in the database IntOGen along with the Global Alliance for Genomics and Health. For localized tumors, she described Cancer Genome Interpreter, a platform that supports the identification of therapeutically actionable genomic alterations in tumors. Dr. Sean Bendall (Stanford University, CA, USA) presented data on mass cytometry as a novel and revolutionary high-throughout tool for single cell proteomics, permitting the examination of multiple parameters in normal human bone marrow or leukemic cells [6]. Using single-cell mass cytometry data analysis with an in-house designed algorithm (Wanderlust) he could produce trajectories predictive of the cellular developmental path in human B-cell lymphopoiesis [7]. He then presented how this can be used to identify developmentally dependent predictors of relapse in diagnostic B-cell precursor acute lymphoblastic leukemia. To close the session, Dr. Carlo Maley (Arizona State University, Tempe, USA) gave the keynote lecture and explained how resources and hazards in tumors select for cell migration and metastasis [8]. He formulated several open questions on cancer evolution such as how to better sample tumors, the relationship between evolution *versus* ecology index and the best predictor of mutation rate. He also introduced ideas about how to manage ITH and suggested an adaptive therapy for cancer treatment, whereby more proliferative but less aggressive clones are not completely eliminated and are used to keep more dangerous populations under control. This implies that after the induction phase of treatment, consolidation chemotherapy should be proportional to the tumor size, in an attempt to maintain the tumor at a stable size and not to completely eradicate it. Therefore, therapeutic strategies should evolve in response to tumor adaptation.

## ITH IN HEMATOPOIETIC MALIGNANCIES

The session was dedicated to ITH in hematologic malignancies, with outstanding talks on the cell-of-origin and the genomic landscape of acute myeloid leukemia (AML) and chronic lymphocytic leukemia (CLL). Dr. John Dick (Sick Children’s Hospital at the University of Toronto, Canada) presented data on a 17-gene leukemia stem cell (LSC) core signature (LSC17) as an independent prognostic factor to predict survival in AML. The LSC17 signature allows for a better stratification of patients receiving induction therapy for whom relapse often occurs, and can be used in diagnosis risk-stratification in AML [9]. He also discussed the nature of the leukemia/relapse-initiating cell in AML; the LSC at relapse can easily differ from that at diagnosis. Importantly, pre-leukemic HSCs carrying DNMT3A mutations can be found at diagnosis, even in the absence of the recurrent driver-initiating event, and be maintained at relapse. Finally, Dr. Dick described cases of clonal hematopoiesis of indeterminate potential either developing (AML+) or not developing (non AML) overt leukemia. He showed that these groups present different mutation types, and oncogenic mutations are found in cases of AML+. Focusing on genetic and phenotypic heterogeneity in AML, Dr. Elli Papaemmanuil (The Memorial Sloan Kettering Cancer Center in New York, USA) presented the revised genomic classification for AML based on cancer gene driver and passenger mutations, and reported the emergence of three novel heterogeneous genomic categories added to the pre-existing classifications: i) chromatin-spliceosome; ii) p53-aneuploidies; and iii) IDH2^R172^ mutations. Her lecture covered topics such as the mutation acquisition order, mutational co-occurrence, mutual exclusivity and deterministic nature of the mutations [10]. She also discussed how to implement precision medicine in AML patients based on banked genomic-clinical data [11]. Finally, Dr. Xosé Puente (University of Oviedo, Spain) provided insights into the genomic architecture of B-CLL. Mature B-cell and B-CLL integrative analysis, constructed with WGS and WES data, as well as RNA sequencing, copy number variation (CNV) and DNA methylation, revealed different B-CLL molecular signatures and provided evidence about recurrent and non-recurrent driver genes [12]. The majority of the genes were mutated at low frequency (sub-clonal), with the most common alterations affecting genes for immunoglobulins, DNA damage response, splicing, NF-kB and Notch pathways. Notably, the number of driver alterations correlated with patient clinical behavior.

## ITH IN SOLID TUMORS

Most ITH results have been acquired by the study of solid tumours. Dr. Mario Suvà (Massachusetts General Hospital and Harvard Medical School, Boston, USA) presented data on single-cell RNA-sequencing of oligodendrioglioma. Six patients were assessed and an in-house written algorithm was used to infer CNV and tumor architecture. While most cancer cells seemed to have transcriptional signatures reminiscent of oligodendrocytes and astrocytes, a few cells within the bulk tumor showed a transcriptome profile resembling a neural stem cell phenotype and cell proliferation expression programs. These cancer stem cells would be responsible for fueling tumor growth [13]. Dr. Suvà also introduced single-cell analysis to comprehend the molecular differences between oligodendrioglioma and astrocytoma cancer cells. On the same topic of brain tumors with dismal prognosis, Dr. Chris Jones (Institute of Cancer Research, Sutton, UK) reported on pediatric high-grade diffuse intrinsic pontine glioma (DIPG). No pre-treatment material was available to study this subgroup until a sophisticated biopsy procedure was implemented some years ago. Molecular studies revealed that point mutations in several histone-coding genes define different clinical subgroups with different prognoses and phenotypes [14]. He also speculated on how cooperation between cancer subclones could promote infiltration, migration and invasion in DIPG. Dr. Marco Gerlinger (Institute of Cancer Research, Sutton, UK) discussed precision medicine in primary renal carcinoma. Multi-region genome and RNA-sequencing profiling revealed a branched evolution of the tumor, with an abundance of subclones with independent branching evolution [15]. He anticipated the difficulty to target subclonal somatic activating mutations of mTOR not present in the tumor trunk. He also presented data on ITH in gastro-oesophageal adenocarcinoma. Targeted sequencing studies revealed a median of three mutations/patient and high chromosomal instability with copy number alterations (CNA) heterogeneous among the sequenced regions. Overall, he emphasized the complexity of the tumor genomic landscape and also the drawbacks of combined polytherapy to target multiple drivers. Dr. Peter Dirks (Sick Children’s Hospital, Toronto) discussed how quiescent Sox2+ cells drive hierarchical growth and relapse in a subgroup of sonic hedgehog-dependent medulloblastoma. He showed how drugs targeting this pathway, such as Smoothened inhibitors (SMOi), are ineffective because Sox2^+^ cells are resistant to SMOi, which only shrink the tumor bulk while leaving a reservoir of self-renewing cells responsible for relapse [16]. He also suggested that enforcing glioblastoma cells to neuronal lineage commitment would decrease ITH, a type of “differentiation therapy”. Dr. Joan Seoane (Vall d’Hebron Institute of Oncology, Barcelona, Spain) presented an update on research on glioblastoma, a brain tumor in which tumor DNA detection by liquid biopsy of cerebrospinal fluid (CSF) has been recently reported to be very informative for clinical interventions. He described a complex heterogeneity at the level of stromal cells, genome and epigenome. He reinforced the use of digital PCR to confirm sequencing results from longitudinal sampling, especially since many mutations found at relapse are commonly undetectable at diagnosis due to very low variant allele frequency. Dr. Alberto Bardelli (University of Torino, Italy) summarized data from the phase 2 clinical trial HERACLES, aimed at assessing dual-targeted therapy with trastuzumab and lapatinib in treatment-refractory HER2-positive metastatic colorectal cancer [17]. He also illustrated the advantages of transrenal DNA as a diagnostic tool to monitor MRD and follow up cancer patients, and the need for new technologies to overcome DNA fragmentation upon infiltration. Finally, Dr. Eric Holland (Fred Hutchinson Cancer Research Center, Seattle, USA), presented how big data visualization identifies the multidimensional molecular landscape of human gliomas. His group found that CNV and single nucleotide alterations across the genome are quite different, generating three main glioma clusters [18]. He also introduced Oncospace, an open source application for disease data sets across multiple cancer types, which allows researchers to discover novel patterns and relationships between clinical and molecular data.

## DEALING WITH ITH IN THE CLINICAL PRACTICE

The meeting ended with a roundtable discussion between speakers and the audience moderated by Dr. Josep Tabernero (Vall d’Hebrón Institute of Oncology, Barcelona, Spain). The benefit of liquid biopsy as a surrogate diagnostic tool when tumor biopsy is not accessible was discussed. Liquid biopsies can retrieve either plasma cell-free circulating DNA (cfDNA) or CTCs [19]. cfDNA can be used to quantify tumor burden, and cfDNA in blood or CSF can be sequenced since its mutational spectrum frequently mirrors that of the tumor of origin. However, it is uncertain whether liquid biopsies phenocopy the malignant phenotype and more investigation is needed to evaluate this. Although attempts to translate the value of cfDNA to the clinical setting are ongoing, the use of liquid biopsies in the clinics awaits approval from regulatory bodies.

The presence of co-existing diverse sub-clones carrying different driver and passenger mutations makes the treatment selection process difficult. Polytherapy can exhaust patients due to excessive adverse effects, and access to new drugs is occasionally delayed by approval and reimbursement bodies. In the setting of pediatric tumors, pediatric oncologists employ therapeutic drugs designed for adults in children but these are not effective. Therefore, oncologists managing neuroectodermal/brain tumors claim the need to think more outside the box. Thinking from a new perspective is necessary for clinicians to introduce new drugs into clinical trials and to explore risky and new treatments.

Finally, the need for crosstalk between researchers and physicians was highlighted. The feeling was that there is a lack of communication to translate laboratory research to clinical practice and *vice versa*. How would it be possible to establish a researcher-physician interaction model? Apart from thinking differently, the main demand was the usage of public resources to share genomic and clinical data.

## CONCLUDING REMARKS

ITH is a major factor contributing to cancer outcome, therapeutic failure and drug resistance. Importantly, ITH does not simply reflect genomic diversity but also variation in epigenetic mechanisms and tumor microenvironment. In spite of recent advances in the ITH field, such as MRS, liquid biopsies, single-cell approaches, increased depth and purity of sequenced material and more sophisticated bioinformatics tools, many key questions remain. For example, the impact of mutations occurring in non-coding regions, the accuracy of evolutionary trajectories reconstructed from genomic data modeling without considering epigenomic and microenvironmental components, the mechanisms of tumor cells to evade the immune system and the viability of precision medicine based on individual genomic make-up in the clinical practice. In this regard, cancer evolutionary therapeutics has only just begun [20] started. Recommendations for future research are to better explore the temporal and spatial dynamics of clones in human tumors. Also, the use of computational, pathological and imaging approaches to better describe the tumor microenvironment is in high demand. In the interest of the patients, these tools should be easily implemented in the clinics. Finally, current educational programs on *in silico* biology should be offered to oncology trainees. Understanding the dynamic evolution of tumors is fundamental for drug development and novel clinical trial designs.
